# Identification of Phenolic Compounds and Evaluation of Antioxidant and Antimicrobial Properties of *Euphorbia Tirucalli* L.

**DOI:** 10.3390/antiox3010159

**Published:** 2014-03-17

**Authors:** Keline Medeiros de Araújo, Alessandro de Lima, Jurandy do N. Silva, Larissa L. Rodrigues, Adriany G. N. Amorim, Patrick V. Quelemes, Raimunda C. dos Santos, Jefferson A. Rocha, Éryka O. de Andrades, José Roberto S. A. Leite, Jorge Mancini-Filho, Reginaldo Almeida da Trindade

**Affiliations:** 1 NIPA-Nucleus of Applied Immunoparasitology, Federal University of Piauí, Parnaíba 64202-020, Brazil; E-Mail: kelinemed@gmail.com; 2 Department of Food and Nutrition, Federal Technological Institute of Piauí, Teresina 64001-010, Brazil; E-Mails: alessandro@ifpi.edu.br (A.L.); jurandy@ifpi.edu.br (J.N.S.); larisages@hotmail.com (L.L.R.); 3 Biodiversity and Biotechnology Research Center, Biotec, Federal University of Piauí, Parnaíba 64202-020, Brazil; E-Mails: adriany1210@gmail.com (A.G.N.A.); pquelemes@gmail.com (P.V.Q.); jrsaleite@gmail.com (J.R.S.A.L.); 4 Master Degree Program in Biotechnology, Federal University of Piauí, Parnaíba 64202-020, Brazil; E-Mails: raimundaphb@hotmail.com (R.C.S.); jeffersonkalel@hotmail.com (J.A.R.); erykaandrades@hotmail.com (E.O.A.); 5 Faculty of Pharmaceutical Sciences, University of São Paulo, São Paulo 05508-900, Brazil; E-Mail: jmancini@usp.br; 6 Department of Toxicological and Clinical Analysis, Faculty of Pharmacy, Federal University of Rio de Janeiro, Rio de Janeiro 21941-902, Brazil

**Keywords:** antimicrobial activity, antioxidant activity, *Euphorbia tirucalli* L., phenolic compounds

## Abstract

Bioactive compounds extracted from natural sources can benefit human health. The aim of this work was to determine total phenolic content and antioxidant activity in extracts of *Euphorbia tirucalli* L. followed by identification and quantification of the phenolic compounds, as well as their antibacterial activities. Antioxidant activities were determined by DPPH and ABTS^•+^ assay. Identification of phenolic compounds was performed using high-performance liquid chromatography (HPLC), and antimicrobial activities were verified by agar dilution methods and MIC values. Total phenolic content ranged from 7.73 to 30.54 mg/100 g gallic acid equivalent. Extracts from dry plants showed higher antioxidant activities than those from fresh ones. The DPPH EC_50_ values were approximately 12.15 μg/mL and 16.59 μg/mL, respectively. Antioxidant activity measured by the ABTS method yielded values higher than 718.99 μM trolox/g for dry plants, while by the Rancimat^®^ system yielded protection factors exceeding 1 for all extracts, comparable to synthetic BHT. Ferulic acid was the principal phenolic compound identified and quantified through HPLC-UV in all extracts. The extracts proved effective inhibitory potential for *Staphylococcus epidermidis* and *Staphylococcus aureus*. These results showed that extracts of *Euphorbia tirucalli* L. have excellent antioxidant capacity and moderate antimicrobial activity. These can be attributed to the high concentration of ferulic acid.

## 1. Introduction

*Euphorbia tirucalli* L., belonging to the family of Euphorbiaceae, is a very popular plant in Northern and Northeastern Brazil. It is widely used as a traditional and natural biomedicine, not only in Brazil, but also in several other tropical countries where it is well adapted to the climate. In Brazil, it is also popularly known as avelóz, cachorro-pelado, árvore-do-lápis, cega-olho, or espinho-italiano [1]. It has been used against several kinds of diseases, infectious and non-infectious, such as syphilis, asthma, rheumatism [2], chancre, and some types of cancer [3], mainly due to the pharmacological properties of its latex. Knowledge about this plant stems mostly from studies of its latex [4], and, to the best of our knowledge, there are no reports in the literature describing systematic experiments characterizing other extracts of *Euphorbia tirucalli* L. with respect to antioxidant properties and identification of subcomponents related to that capacity.

Free radicals and other oxidants are molecules formed by the body’s normal metabolism that can cause harm if not well-controlled. However, the body produces other molecules to destroy the oxidants and defend itself from this damage. These molecules are known as antioxidants [5]. In some situations, though, an unbalanced state between the production and elimination of oxidants may occur [6]. This is known as oxidative stress, where non-eliminated or non-neutralized oxidants can damage many kinds of molecules. For instance, oxidation of lipids leads to lipid peroxidation and membrane disruption, of proteins and enzymes causes fragmentation and loss of biological activity, and of DNA results in random cross linking, leading to cell death [7]. Secondary effects of oxidative stress are believed to be the molecular basis in the development of chronic and degenerative diseases, such as cancer, neurodegenerative disorders, cardiovascular diseases, diabetes, and autoimmune disorders [5,8].

Several studies have strongly evidenced a correlation between polyphenols, mainly those found in herbs, spices, cereals, fruits, and vegetables, and their high antioxidant capacities. Others have indicated that their regular consumption can help to significantly reduce the incidence of diseases linked to oxidative stress, because polyphenols act as potent free radical scavengers [9,10]. Given that, there has been a significant increase in research focused on natural antioxidants, not only for their health benefits, but also as replacements for synthetic additives in food and pharmaceuticals. By definition, antioxidants are substances that counteract free radicals and prevent the damage caused by them. These molecules can greatly reduce the damage caused by oxidants, neutralizing them before they react with biologic targets, either by preventing chain reactions or by preventing the activation of oxygen to highly reactive products [5,11].

**Figure 1:**
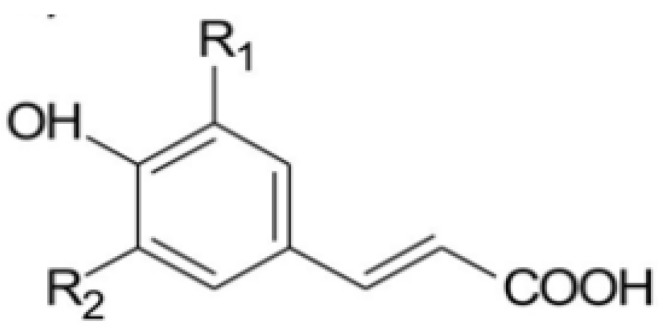
Chemical structure of hydroxycinnamic acid [14].

The term “antioxidant” encompasses a vast array of substances which are divided into exogenous and endogenous antioxidants, and can be classified into two major groups, enzymatic and non-enzymatic antioxidants [5]. The endogenous antioxidants include enzymes, low-molecular-weight molecules, and enzyme cofactors. Exogenous antioxidants can be classified into various classes [12], of which polyphenols are the largest. The secondary metabolism of plants is responsible for the production of phenols and polyphenols, which contain multiple structures, but commonly one or more aromatic rings, respectively [13]. These molecules are classified as flavonoids and phenolic acids [5,11]. Flavonoids and their glycosides are complex molecules, such as catechins, proanthocyanidins, anthocyanins, and flavonols, containing multiple aromatic rings. Among phenolic acids are the hydroxycinnamic acids, the most common compounds found in fruits and seeds, and the monophenols, which possess a single benzene ring (Figure 1). Caffeic and ferulic acids are included in this class [13]. Hydroxybenzoic acids, such as ellagic acid and gallic acid, are the second group of phenolic acids, but their content in plants are very low [5].

To date, identification of the phenolic compounds and resultant antioxidant activities in *Euphorbia tirucalli* L. extracts have not been performed. Therefore, this study was devised to evaluate the antioxidant potential in the aerial part of the plant using various assays, such as free radical scavenging DPPH^•^, ABTS^•+^, and Rancimat methods, followed by the identification of phenolic compounds through high-performance liquid chromatography (HPLC). In addition, the biological activity and pharmacological potential were determined by testing the extracts on different pathogenic bacterial strains to evaluate their inhibitory effects on the growth of these microorganisms.

## 2. Experimental Section

### 2.1. Equipment, Chemicals, and Reagents

HPLC was performed using a Shimadzu Class-VP HPLC system (Tokio, Japan), a computer-controlled system with LC solution Release 1.24 SP1 (Shimadzu Corporation, Kyoto, Japan) and a CBM-20A VP system controller (Shimadzu Corporation, Kyoto, Japan). Other accessories included a Shimadzu DGU-20A degasser, an FCV-20AL mixer, two LC-6AD Shimadzu liquid chromatography pumps, an SIL-10F auto-injector, a CTO-20 column oven, and an SPD-M20A VP diode array detector, all from Shimadzu Corporation, Kyoto, Japan.

The following phenolic standards were obtained from Sigma Chemical Co. (St Louis, MO, USA): gallic acid, vanillic acid, benzoic acid, quercetin, ferulic acid, caffeic acid, *p*-coumaric acid, catechin, ellagic acid, and chlorogenic acid. HPLC-grade acetonitrile and acetic acid were used for HPLC, whereas all other reagents were analytical grade.

Aerial parts of the *Euphorbia tirucalli* L. were collected from Teresina, State of Piauí, Brazil, at 0°04′20.16′′ S and 42°45′39.67′′ W, between November and December, 2011. They were identified by Professor Ivanilza Moreira de Andrade, PhD, Laboratory of Taxonomy, Federal University of Piauí. A voucher specimen was deposited (protocol number 27939) in the Herbarium Graziela Barroso, at the Center of Natural Sciences, Federal University of Piauí, Piauí, Brazil.

### 2.2. Extract Preparation

The *Euphorbia tirucalli* L. samples were analyzed in the Laboratory of Food Analysis at the Federal Institute of Piauí. They were divided in two groups, the first of which was washed with distilled water and dried in the oven for 17 h at 40 °C with closed air circulation. Dry samples were powdered using an analytical mill, and stored at −20 °C until extract preparation. The second group was maintained at room temperature then powdered and stored at −20 °C. Separate extractions of 20 g of fresh sample and 10 g of dry sample were prepared by stirring at 10 rpm in either acetone (200 mL) or 80:20 methanol:water (200 mL) for 24 h. After filtering using # 4 Whatman paper filters (20–25 μm particle retention) under vacuum, the extracts were stored in amber flasks. The four solutions will be referred to as: hydromethanolic fresh (FHE), acetonic fresh (FAE), hydromethanolic dry (DHE), and acetonic dry (DAE) extracts.

### 2.3. Determination of Total Phenolics

Total phenolic content of the extracts was measured using Folin–Denis reagent [15], adapted by Lima [16]. First, either 0.5 mL of the extract or an appropriate gallic acid standard was added to 8.0 mL deionized water. To this, 0.5 mL of Folin–Denis reagent was added and mixed for 3 min, after which 1 mL of saturated Na_2_CO_3_ solution was added and mixed. After dark incubation for 60 min at room temperature, absorbance was measured at 720 nm *versus* a prepared blank. The blank consisted of 0.5 mL 50% (v/v) methanol. Gallic acid at 5, 10, 20, 40, 60, and 80 μg/mL in 50% (v/v) methanol was used as a standard by which a calibration curve was obtained daily. Total phenolics was expressed as mg gallic acid equivalent (GAE)/100 g of dry sample. All samples were analyzed in triplicate.

### 2.4. Evaluation of Antioxidant Activity: DPPH^•^ Method

The colorimetric 1,1-diphenyl-2-picrylhydrazyl (DPPH) radical scavenging assay was carried out according to the protocol described by Blois [17] and adapted by Brand-Williams *et al.* [18]. Briefly, 0.5 mL of ethanol containing different concentrations (2.5, 5, 10, 20, and 40 μg/mL) of each extract (v/v) was mixed with 1.5 mL of DPPH^•^ ethanol solution (6 × 10^−5^ M). The DPPH^•^ solution served as a control. Absorbance was measured at 517 nm after the mixture was kept at room temperature for 2, 5, 10, 20, and 30 min. Antioxidant activity, expressed as percentage of DPPH radical scavenging activity (RSA), was calculated as follows, Equation 1:

% RSA = [(Abs blank − Abs sample)/Abs blank] × 100%
(1)

where Abs blank is the absorbance of the control and Abs sample is the absorbance of the DPPH solution with the extract.

Extract concentrations providing 50% inhibition (IC50) were found by interpolation from the plot of inhibition percentage. All tests were run in triplicate, and the average value calculated.

### 2.5. Evaluation of Antioxidant Activity: ABTS^•+^ Method

The ABTS assay was based on the method described by Re *et al.* [19]. Briefly, 7 mM of ABTS was mixed with 140 mM of potassium persulfate an in aqueous solution. The mixture was incubated at room temperature for 12 h, allowing formation of the radical ABTS^•+^. The blue-green ABTS^+^ solution was diluted with ethanol until the absorbance reached 0.700 ± 0.020 at 734 nm. Then, either 40 μL of Trolox^®^ standard (concentrations ranging from 50 to 800 μM) or extracts were mixed with 1960 μL of ABTS^•+^ solution and kept at room temperature. The decrease of absorbance was monitored at 734 nm after 2, 5, 10, 20, and 30 min. Due to a gradual decrease in absorbance of the ABTS stock solution (2%/h), blanks were recorded for each measurement, and consisted of 40 μL of ethanol in 1960 μL of radical solution. Results were expressed in μM Trolox/g sample.

### 2.6. Evaluation of Antioxidant Activity: Rancimat Method

Antioxidant activities in oil oxidations of the extracts were found using a Rancimat model test (Metrohm model 873, Herisan, Switzerland), at 110 °C, a slight adaptation of Jardini and Mancini-Filho [20]. The air flow rate was fixed at 20 L/h. Five grams of antioxidant-free soybean oil (Dureino^®^ industry) was used as the lipid substrate. Concentrations of the test extracts were 0.01% and 0.02% (w/w) on a dry weight basis. Butylated hydroxytoluene (BHT) was used as a comparative synthetic antioxidant. The effects of the samples on retarding oil oxidation, expressed as the protection factor (PF), was calculated as follows, Equation 2:

PF = IP_antiox_/IP_contrl_ (2)

where IP_antiox_ and IP_contrl_ are the induction periods (IPs) of oil oxidation with and without antioxidant, respectively.

### 2.7. Identification and Quantification of Phenolic Compounds Using HPLC

This methodology was adapted from Dóbias *et al.* [21], and Mira *et al.* [22]. The wavelength selected for the detection of phenolic acids was 253 nm while the column oven temperature was set to 35 °C. Phenolic acid standards used for the detection and identification of peaks were gallic acid, vanillic acid, benzoic acid, quercetin, ferulic acid, caffeic acid, *p*-coumaric acid, catechin, ellagic acid, and chlorogenic acid. An auto injector was used to inject 40 μL of the extract onto the HPLC column while the eluent flow rate was set to 1.0 mL/min. Aqueous acetic acid was used as mobile phase A and acetonitrile as mobile phase B. Elution consisted of the following gradient: 80:20 A:B for 5.0 min, ramped to 50:50 over 5–7 min, then set to a constant 28:72 for 7–20 min. The column was washed with 100% acetonitrile for 30 min and reconditioned with 20% acetonitrile for 20 min between runs. Each standard was recorded and stored in the HPLC spectrum library. The criteria for identification of *Euphorbia tirucalli* L. compounds were established by comparing the retention time and spectra of unknown compounds with those in the HPLC library of standards. The purity of the peaks was determined to ensure identification. For close peaks, an integration program in the HPLC software was used to split them and produce data for calculations. Phenolic acids contained in the extracts were quantified by an external standard method. The concentration selected for identifying phenolic acid in all samples was 0.001%, whereas the calibration curves were constructed at 10, 50, 200, and 325 μg/mL. Calibration curves were built by plotting the phenolic acid peak at 253 nm against the phenolic acid standard. Data were analyzed by linear regression.

### 2.8. Antibacterial Assay

Antibacterial assays were conducted using 10 mL of each extract submitted to solvent evaporation over 10 h in a vacuum concentrator (Centrivap^®^, Kansas City, MO, USA) after which 5 and 10 mg of each dried extract was resuspended in 1.0 mL of 15% dimethyl sulfoxide (DMSO) in sterile distilled water, reaching final concentrations of 5000 μg/mL and 10,000 μg/mL for further testing.

Extracts were tested for activity against *Staphylococcus aureus* (ATCC 29213), *Staphylococcus epidermidis* (ATCC 12228), *Enterococcus faecalis* (ATCC 29212), *Escherichia coli* (ATCC 25922), and *Pseudomonas aeruginosa* (ATCC 27853). Bacteria were spread over Muellen-Hinton agar (DIFCO^®^) and aerobically incubated for 24 h at 37 °C, after which they were collected and suspended in sterile saline [0.85% NaCl (w/v)] until an absorbance of 0.080 to 0.100, at 625 nm, representing approximately 1 × 10^8^ CFU/mL. This bacterium solution was used for the following procedures.

#### 2.8.1. Agar Dilution

This method is recommended by the NCCLS [23] with some adaptations from Maia-Araújo *et al.* [24]. Approximately 1 × 10^8^ CFU was inoculated *per spot* for each microorganism followed by 60 μL of extract with a concentration of phenolic compounds between 5 and 10 mg/mL. The plates were incubated for 24 h at 37 °C in ambient conditions. A 15% DMSO solution (v/v) was used as a control. All experiments were performed in triplicate.

#### 2.8.2. MIC through Microdilution Method

Minimum inhibitory concentrations (MICs) were measured through microdilution of the Mueller–Hinton broth following the recommendations of the NCCLS [25] and Ostrosky *et al.* [26]. Each extract was diluted to 6.25 to 800 μg/mL by dispensing 16 μL of extract into each of the 96 wells of a standard microtiter tray containing 179 μL of Mueller-Hinton broth followed by 2-fold dilutions. The standardized inoculum (5 μL) was added to give a final concentration of 5 × 10^5^ CFU/mL, reaching a final volume of 100 μL in each well. As a positive control, 95 μL and 5 μL of bacteria inoculums were added to a well. After ambient incubation at 37 °C for 24 h, MIC was recorded as the lowest concentration of extract inhibiting visible growth compared to the control.

### 2.9. Statistical Analysis

Means and standard deviations (SDs) were obtained for all experiments. Results were analyzed using a one-way analysis of variance (ANOVA) using a beta version of ASSISTAT software version 7.8 (2012) (Federal University of Campina Grande City, Campina Grande, Brazil) in order to assess the differences between the types of extracts. When statistically significant differences were found (*p* < 0.05), *Tukey* tests were performed.

## 3. Results and Discussion

Phenolic compounds are molecules widespread in the plant kingdom. These compounds are produced in the secondary metabolism of fruits, herbs, and vegetables [14]. Several studies have indicated beneficial health effects of increasing consumption of these foods. In plants, they can contribute to growth, reproduction, and pigmentation, whereas in foods, they are responsible for maintaining oxidative stability [14]. They also exert influence on flavor and astringency [27]. In the pharmacological or food industries, the discovery or even description of new sources of bioactive compounds are always welcome, principally in view of replacing synthetic compounds or overcoming pharmaceutical dilemmas, such as the resistance of some microorganisms to antibiotics. In this context, the rationale of our experiment is to screen and describe the antioxidant activities of various extracts of *Euphorbia tirucalli* L., as such information is not currently reported in the literature. The use of dry and fresh aerial parts of *Euphorbia tirucalli* L., and solvents of different polarities such as water, methanol, and acetone, target extraction of compounds from different compartments in the plant.

### 3.1. Determination of Total Phenolics

Table 1 shows that the total phenolic content found in the extracts of *Euphorbia tirucalli* L. was 7.7 and 30.5 mg GAE/100 g sample and 9.4 and 27.6 mg GAE/100 g sample for FAE and DAE, and FHE and DHE, respectively. Higher phenolic concentrations were found in dry extracts as compared to fresh ones (*p* < 0.05).

**Table 1:** Total phenolic content present in different extracts of aerial part of *Euphorbia tirucalli* L., reported in gallic acid equivalents (GAE).

Samples	Total Phenolics (mg GAE/100 g Sample) *
Dry acetonic extract	30.54 ± 0.64 ^a^
Fresh acetonic extract	7.73 ± 1.21 ^b^
Dry hydromethanolic extract	27.6 ± 2.34 ^a^
Fresh hydromethanolic extract	9.47 ± 0.65 ^b^

* Values are shown as mean and standard deviation (*n* = 3); Different letters (a, b) mean significant differences (*p* < 0.05).

### 3.2. Evaluation of Antioxidant Activity: DPPH^•^ Method

Table 2 shows that all extracts exhibited significantly higher antioxidant activity than the control, catechin (10.78 μg/mL) (*p* < 0.05), but dry extracts (DAE and DHE) showed lower EC_50_ values, 13.95 μg/mL and 12.15 μg/mL, respectively.

**Table 2:** Percentage of Radical Scavenging Activity (% RSA), and EC_50_ of different extracts of aerial part of *Euphorbia tirucalli* L., determined by DPPH method.

Samples	Concentrations % RSA					
	2.5 μg/mL	5.0 μg/mL	10 μg/mL	20 μg/mL	40 μg/mL	EC_50_ (μg/mL)
DAE	11.42 ± 2.38	25.65 ± 2.99	58.57 ± 5.02	83.53 ± 1.81	85.03 ± 1.15	13.95 ± 0.33 ^a,b^
FAE	13.93 ± 5.99	19.38 ± 5.91	38.11 ± 8.14	77.08 ± 0.84	96.23 ± 3.50	15.91 ± 1.86 ^a,c^
DHE	20.42 ± 0.9	24.93 ± 0.89	55.81 ± 0.60	87.64 ± 0.68	95.13 ± 0.13	12.15 ± 0.09 ^b,d^
FHE	9.06 ± 2.27	17.74 ± 2.66	43.49 ± 3.13	74.05 ± 2.60	94.14 ± 1.69	16.51 ± 0.53 ^c^
Catechin	19.9 ± 2.83	32.74 ± 1.44	60.11 ± 5.57	88.90 ± 1.30	91.90 ± 0.16	10.78 ± 0.57 ^d^

* Values are shown as mean and standard deviation (*n* = 3). DAE: Acetonic dry extract; FAE: Acetonic fresh extract; DHE: Hydromethanolic dry extract; FHE: Hydromethanolic fresh extract. Superscript lower case letters (a–d) within a column means comparison among each one of different extracts and catechin. Different letters share significant differences at *p* < 0.05.

In general, the best results in terms of either the phenolic content or antioxidant activity were obtained from dry plant extracts. The effects of drying fresh herbs (more specifically rosemary, oregano, sage, marjoram, basil, and thyme) before extraction of their constituents [28] have been well recognized and reported in the literature. Fawole *et al.* [29] analyzed different medicinal plants from South Africa, in which all tested methanolic extracts showed EC_50_ values ranging from 5.0 μg/mL to 10.0 μg/mL. In comparison, *Euphorbia tirucalli* L. showed slightly less antioxidant activity. Another study, performed by Akinmoladun *et al.* [30] used medicinal plants from Nigerian Indigenous. The reported RSA was 21.0% to 88.0%, but in that case, an extract concentration of 300 μg/mL was used. The extract from *Euphorbia tirucalli* L. demonstrated an RSA above 85.0% using a maximum concentration of 40 μg/mL. These results confirm that *Euphorbia tirucalli* L. has high antioxidant potential. Melo *et al.* [31] analyzed other Euphorbiaceae species, such as *Croton blanchetianus* Baill and *Jatropha mollissima* (Pohl) Baill, finding an IC_50_ of 94.0 and 55.0 μg/mL, respectively. These values are comparable to results found for *Euphorbia tirucalli* L.

### 3.3. Evaluation of Antioxidant Activity: ABTS^•+^ Method

Table 3 shows the antioxidant activity evaluated by the ABTS^•+^ method. Again, dry extracts showed higher antioxidant activity. After 30 min, dry extracts showed Trolox Equivalent Antioxidant Capacity (TEAC) values of 716 and 718 μM Trolox/g sample for DAE and DHE, respectively. The reaction was saturated after 10 and 20 min for FHE and FAE, respectively.

As no previous studies have reported the specific antioxidant activity of other species of *Euphorbia* sp., and taking into consideration results of antioxidant activities found in other medicinal plants, the results found in this study show a strong antioxidant potential in this plant family, particularly if first dried. Gulati *et al.* [32] evaluated the antioxidant activity by ABTS^•+^ methodology in twelve medicinal plant extracts, including some from Euphorbiaceae, and found maximum TEAC values of 374 μg/mL for ethanolic extracts, using ascorbic acids and BHT as controls. In *Euphorbia tirucalli* L., extracts of dried samples exhibited TEAC values above 500 μg/mL after only 2 min of reaction, proving high antioxidant potential. One fact that must be considered from Table 3 is that after two minutes of reaction, all extracts continued neutralizing free radicals, consequently increasing TEAC values. Some authors prefer to analyze TEAC at 2 min while others prefer reaction times up to 60 min, explaining that antioxidants in the extracts continue to neutralize free radicals present in the solution [33]. Song *et al.* [34] studied fifty-six Chinese medicinal plants and found TEAC values to vary widely according to the type of plant, however, an optimal correlation between total phenolic quantification and antioxidant activity was found, indicating that these molecules are responsible for that activity. With respect to *Euphorbia tirucalli* L., it was found that higher phenolic content in the extracts coincided with higher antioxidant activity.

**Table 3:** Antioxidant capacity by TEAC (Trolox Equivalent Antioxidant Capacity) of different extracts of aerial part of *Euphorbia tirucalli* L*.*, using ABTS^•+^ method reported as μM TROLOX/g sample.

Samples	TEAC Value—μM Trolox/g Sample				
	2 min	5 min	10 min	20 min	30 min
DAE	45,344 ± 8513 ^a^^,A^	54,566 ± 1,0333 ^a^^,A^	59,566 ± 12,662 ^a^^,A^	67,566 ± 15,377 ^a^^,A^	71,677 ± 17,160 ^a^^,A^
FAE	7002 ± 488 ^b^^,B^	10,085 ± 419 ^b^^,C^	11,336 ± 474 ^b^^,A^	11,766 ± 555 ^b^^,A^	11,891 ± 300 ^b^^,A^
DHE	52,008 ± 1892 ^a^^,C^	60,344 ± 4525 ^a^^,A^^,C^	64,010 ± 6185 ^a^^,A^^,C^	68,788 ± 7395 ^a^^,A^	71,899 ± 7688 ^b^^,A^
FHE	7502 ± 801 ^b^^,C^	9307 ± 577 ^b^^,A^^,C^	10,224 ± 416^b^^,A^	10,363 ± 947 ^b^^,A^	10,447 ± 1748 ^a^^,A^

* Values are shown as mean and standard deviation (*n* = 3). DAE: Acetonic dry extract; FAE: Acetonic fresh extract; DHE: Hydromethanolic dry extract; FHE: Hydromethanolic fresh extract. Different capital letters (A–C) within the same time (column) differ significantly (*p* < 0.05); different small letters (a, b) mean significant differences within the same extract (line) at *p* < 0.05.

### 3.4. Evaluation of Antioxidant Activity: Rancimat Method

**Figure 2:**
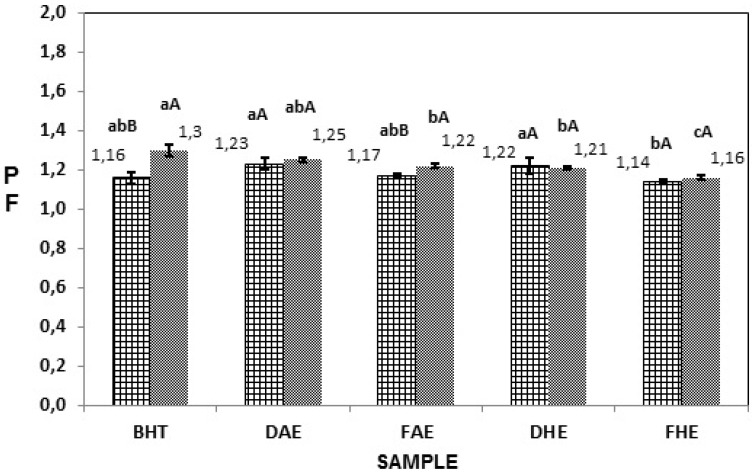
Protection factor (PF) of different extracts of aerial part of *Euphorbia tirucalli L.*, in the concentration of (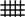) 0.01% (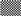) 0.02% using Rancimat method sample. Mean values (*n* = 3, error bars represent standard deviation). BHT: Synthetic Antioxidant Control; FHE: Hydromethanolic fresh extract; DHE: Hydromethanolic dry extract; FAE: Acetonic fresh extract; DAE: Acetonic dry extract. Different capital letters (A–C) differ significantly among the concentrations tested within the same extract (*p* < 0.05); different small letters (a, b) mean significant differences among extracts (*p* < 0.05).

This methodology verified that soybean oil without antioxidant, used as control, oxidized after an IP of 6.4 h. As extracts having antioxidant capacities or synthetic antioxidants are added to soybean oil, the IP rises, and the PF, calculated from difference between synthetic and tested antioxidants, rises above 1.0. Extracts in different concentrations (0.01% and 0.02%), were tested and shown to be effective. See Figure 2. The highest PF values were found in DAE, at 1.23 and 1.25 for concentrations of 0.01% and 0.02%, respectively. The order of stability obtained as a result of the addition of the extracts at 0.01% was BHT > DAE > DHE > FAE > FHE, while at 0.02%, it was BHT > DAE > FAE > DHE > FHE.

Confirming the results of DPPH^•^ and ABTS^•+^ methodologies, the antioxidant potential of extracts from *Euphorbia tirucalli* L. were found to be high when tested in an oil matrix, representing an industrial approach. Very common in the food industry is the use of synthetic antioxidants such as BHT, BHA, or TBHQ in edible vegetable oils, in order to extend shelf life [27]. Such antioxidants help protect them against oxidation. On the other hand, studies have demonstrated possible undesirable health effects of these synthetic additives [35,36]. For this reason, consumers have given preference to natural additives in foods [37]. This shows the importance of continuously seeking other sources of antioxidants, particularly from natural sources. A test commonly used in the food industry is the Rancimat^®^ method.

Beddows *et al.* [38] performed the Rancimat^®^ method on different herbs and spices, finding maximum Pf values of 1.81 using a concentration of 0.2% of thyme. No extracts of *Euphorbia tirucalli* L. obtained PF values higher than BHT (1.16 and 1.30 for concentrations of 0.01% and 0.02%, respectively), but taking into consideration that BHT is a synthetic antioxidant with recognizable antioxidant capacity, extracts with Pf values nearing that of BHT must be considered good protection against oxidation.

### 3.5. Identification and Quantification of Phenolic Compounds Using HPLC

**Figure 3:**
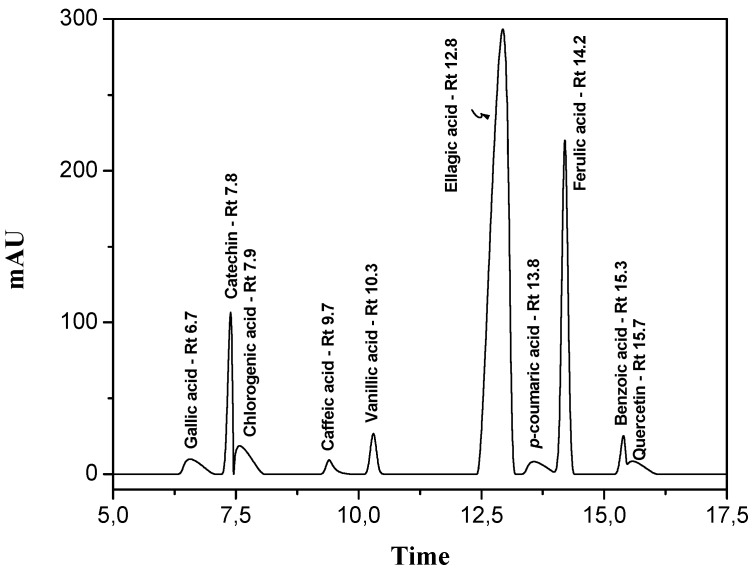
Schematization of the HPLC chromatogram showing the ten standard phenolic acids and their respective retention times used for identification of these compounds in the extracts.

Figure 3 shows the HPLC chromatogram of the standard phenolic acids used for the identification of the compounds in the extracts. Ten standard phenolics were analyzed to determine their retention times under the same conditions as that of the extracts.

**Figure 4:**
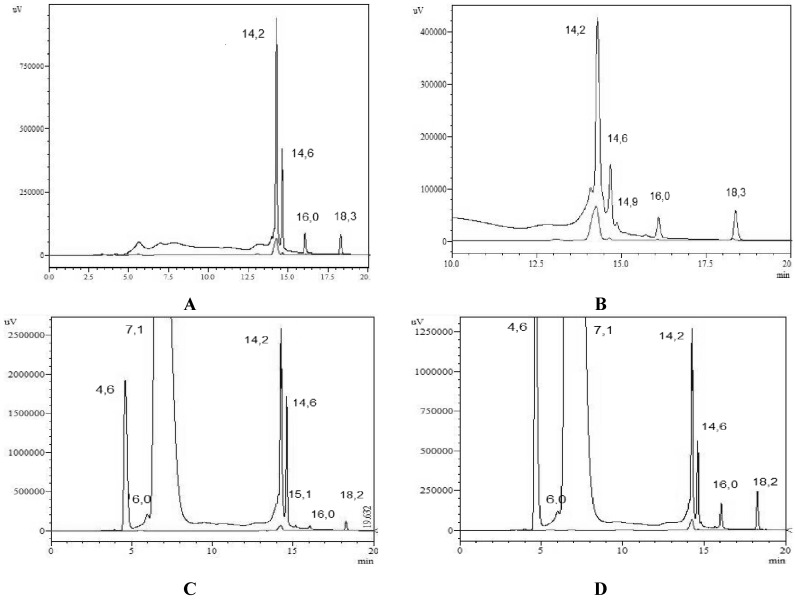
Identification of phenolic compounds by comparison of the BPC (base peak chromatogram) of standard phenolics and different extracts of aerial part of *Euphorbia tirucalli* L., resolved by HPLC system. (**A**) DHE: Hydromethanolic dry extract; (**B**) FHE: Hydromethanolic fresh extract; (**C**) DAE: Acetonic dry extract; (**D**) FAE: Acetonic fresh extract. (Superior line) Extract; (Baseline) Standard ferulic acid.

Under these analytical conditions, ferulic acid was the only phenolic acid identified, with a standard retention time of 14.2 min. Its peak coincided with the more abundant peak in extracts from *Euphorbia tirucalli* L. Chromatographic peaks of dry extracts were more prominent (mAU) than those of fresh extracts, confirming previous results obtained by DPPH, ABTS, and Rancimat^®^ methods. Comparative chromatograms of the extracts and the standards are presented in Figure 4A–D.

The concentration of the principal phenolic compound found in *Euphorbia tirucalli* L. was calculated using a calibration curve obtained from standard ferulic acid (Table 4).

**Table 4:** Quantification of ferulic acid in different extracts from aerial part of *Euphorbia tirucalli* L., by high-performance liquid chromatography (HPLC) expressed in g/100 g sample.

Samples	Ferulic Acid (g/100 g Sample) *
Dry acetonic extract	0.40 ± 0.014 ^a^
Fresh acetonic extract	0.12 ± 0.000 ^b^
Dry hydromethanolic extract	0.59 ± 0.028 ^c^
Fresh hydromethanolic extract	0.08 ± 0.007 ^b^

* Values are shown as mean and standard deviation (*n* = 3).

To identify phenolic compounds present in different extracts from *Euphorbia tirucalli* L., the HPLC/UV-Vis method was adapted from other methodologies found in the literature, where some changes in the elution gradient was used. Phenolic acids contained in the extracts of *Euphorbia tirucalli* L. were identified by comparing retention times to those of standard phenolics. According to Broinizi *et al.* [39], ferulic acid is a strong free-radical scavenger, even in low concentrations. This statement reinforces the assumed relationship between antioxidant activities described in *Euphorbia tirucalli* L. and the presence of this phenolic compound in all the analyzed extracts. As stated before, phenolic acids are divided into two groups: derivatives of hydroxybenzoic acid and those of hydroxycinnamic acid. These molecules are characterized by a benzene ring, a grouping containing one or more carboxylic groups, and a hydroxyl/methoxyl group, which explains the fact that vegetables are strongly associated with antioxidant activity. The ferulic acid belongs to the hydroxycinnamic acid group, which consists of aromatic compounds with three carbon atoms on the side chain. These molecules are more active as antioxidants because they have a double bond in their structure, providing stability for the free radical [14]. Our results are in agreement with studies performed by Ranilla *et al.* [40] which identified in *Chancapiedra*, a plant of family Euphorbiaceae, large quantities of hydroxycinnamic acid (2.9 mg/g sample). In another study performed by Nazemiyeh *et al.* [41], hydroxycinnamic acid was identified in extracts of *Euphorbia petiolata*.

### 3.6. Antibacterial Assay

Table 5 shows the antibacterial activities of the extracts, expressed in size of inhibition zone of bacterial growth. None of the gram-negative bacteria were sensitive to any of the tested extracts; however the gram-positive *S. aureus* and *S. epidermidis* were susceptible. At a concentration of 10 mg/mL, *S. epidermidis* was susceptible in an inhibition zone of 12.8 to 16.0 mm while *S. aureus* was susceptible in an inhibition zone of 13.2 to 13.7 mm. At a concentration of 5 mg/mL, none of the tested strains were sensitive.

**Table 5:** Antibacterial activity of different extracts of aerial part of *Euphorbia tirucalli* L., against pathogenic microorganism strains.

Samples	Extract (10 mg/mL) *			
	FAE	DAE	FHE	DHE
*S. epidermidis*	15.2 ± 0.17	16.0 ± 0.70	12.8 ± 0.17	14.0 ± 0.70
*S. aureus*	13.7 ± 0.55	13.4 ± 0.28	13.2 ± 0.50	13.4 ± 0.26
*E. faecalis*	-	-	-	-
*E. coli*	-	-	-	-
*P. aeruginosa*	-	-	-	-

* Values are shown as mean and standard deviation of inhibition zone in mm (*n* = 3); (-) inhibition zone absence.

*S. aureus* was most affected by FAE, which produced a larger inhibition zone, whereas *S. epidermidis* was most affected by DAE. The MIC was measurable only with respect to *S. epidermidis*. FAE, DAE, and DHE exhibited a MIC of 200 μg/mL while FHE measured 400 μg/mL MIC. All other strains showed no sensitivity to any of the extracts used.

The resistance of pathogens to human and animal drugs is a problem affecting both developed and developing countries. The indiscriminate and uncontrolled use of antibiotics has generated strains of multi-resistant bacteria, causing serious public health problems. This has stemmed increasing research focused on finding new antimicrobial substances [42]. Most plants produce secondary metabolites for self-defense against bacteria, viruses, and parasites. These metabolites have been associated with beneficial medicinal and health effects. Currently, there are many studies exploring plant extracts in order to find those with the best antibacterial activities. For instance, Upadhyay *et al.* [43] analyzed the action of methanolic extracts of leaves and stem bark of *Euphorbia tirucalli* L. on different bacterial strains at concentrations of 2.5, 5, and 7.5 mg/mL showing that strains of *S. aureus*, *P. aeruginosa*, and *E.* *coli* were sensitive to the effects of the tested extracts. These results coincide with those presented here, specifically with those found for *S. aureus*. Upadhyay *et al.* [43] also reported that gram-negative bacteria are more resistant to the actions of the extracts, explaining that this is possibly due to outer membrane lipopolysaccharide (LPS) structures, which hinder toxic and antimicrobial effects. Vale and Orlanda [44] also evaluated the antibacterial activity of fresh and dry ethanolic extracts from aerial parts of *Euphorbia tirucalli* L. at 0.09 mg/mL, showing that dry extracts have no inhibition on bacterial growth, with the exception of *Vibrio parahaemolyticus*. Fresh extracts showed antimicrobial activity against *Salmonella typhi*, *S. aureus*, *Vibrio parahaemolyticus*, *Citrobacter freundii*, and *Serratia odorífera*, whereas *Escherichia coli* and *Pseudomonas aeruginosa* were less susceptible.

Phenolic compounds and polyphenols in plants are toxic products to pathogenic microorganisms, insects, and herbivores. In general, this toxicity is correlated to the position and number of hydroxyl groups in the molecule [45]. The antimicrobial activity found in this study can be attributed to the large quantity of ferulic acids as active constituents in *Euphorbia tirucalli* L., as identified by HPLC.

## 4. Conclusions

Based on our results, it is possible to conclude that the dry extracts showed higher values than fresh extracts, mainly due to their higher concentration of phenolics, possibly caused by dehydration before the extraction process. The methodology used to study the different extracts from *Euphorbia tirucalli* L. allowed identification and quantification of the phenolic compounds, of which ferulic acid was the most prominent. This phenolic compound was the most visible peak of the samples, which were associated with the high antioxidant potential of this plant as analyzed by different and specific methods. Additional studies must be performed for isolation and further characterization of bioactive compounds present in *Euphorbia tirucalli* L., as well as purification and enrichment of ferulic acid. Once the concentration of ferulic acid in this plant is identified, other antimicrobial assays can be performed, such as those for antiparasitic activity. These results justify the use of this plant in traditional medicine to treat infections and injuries, as well as some diseases. Furthermore, our results indicate that “aveloz” shows a good and viable alternative for both the treatment of diseases and a possible replacement for the traditional synthetic antioxidants.

## References

[1] Dutra R.C., Claudino R.F., Bento A.F., Marcon R., Schmidt E.C., Bouzon Z.L., Pianowski L.F., Calixto J.B. (2011). Preventive and therapeutic euphol treatment attenuates experimental colitis in mice. PLos One.

[2] Bani S., Kaul A., Khan B., Gupta V.K., Satti N.K., Suri K.A., Qazi G.N. (2007). Anti-arthritic activity of a biopolymeric fraction from *Euphorbia tirucalli*. J. Ethnopharmacol..

[3] Melo J.G., Santos A.G., Amorim E.L.C., Nascimento S.C., Albuquerque U.P. (2011). Medicinal plants used as antitumor agents in Brazil: An ethnobotanical approach. Evid. Based Complement. Altern. Med..

[4] Freitas C.D.T., Souza D.P., Araújo E.S., Cavalheiro M.G., Oliveira L.S., Ramos M.V. (2010). Anti-oxidative and proteolytic activities and protein profile of laticifer cells of *Cryptostegia grandiflora*, *Plumeria rubra* and *Euphorbia tirucalli*. Braz. J. Plant Physiol..

[5] Ratnan D.V., Ankola D.D., Bhardwaj V., Sahana D.K., Kumar M.N.V.R. (2006). Role of antioxidants in prophylaxis and therapy: A pharmaceutical perspective. J. Control. Release.

[6] Sies H. (1997). Oxidative stress: Oxidants and antioxidants. Exp. Physiol..

[7] Beckman K.B., Ames B.N. (1998). The free radical theory of aging matures. Physiol. Rev..

[8] Pham-Huy L.A., He H., Pham-Huy C. (2008). Free radicals, antioxidants in disease and health. Int. J. Biomed. Sci..

[9] Wang S., Melnyk J.P., Tsao R., Marcone M.F. (2011). How natural dietary antioxidant in fruits, vegetables and legumes promote a vascular health. Food. Res. Intern..

[10] Wootton-Beard P.C., Ryan L. (2011). Improving public health? The role of antioxidant-rich fruit and vegetables beverages. Food. Res. Int..

[11] Azzi A., Davies K.J.A., Kelly F. (2004). Free radical biology—Terminology and critical thinking. FEBS Lett..

[12] Liu R.H. (2004). Potential synergy of phytochemicals in cancer prevention: mechanism of action. J. Nutr. Sci..

[13] Gulçin I. (2012). Antioxidant activity of food constituents: An overview. Arch. Toxicol..

[14] Angelo P.M., Jorge N. (2007). Compostos fenólicos em alimentos: Uma breve revisão. Rev. Inst. Adolfo Lutz.

[15] Swain T., Hills W.E. (1959). The phenolic constituents of *Prunus domestica*. J. Sci. Food. Agric..

[16] Lima A. (2008). Caracterização Química, Avaliação da Atividade Antioxidante *in vitro* e *in vivo*, e Identificação dos Compostos Fenólicos Presentes no Pequi (*Caryocar brasiliense*, Camb.). Ph.D. Thesis.

[17] Blois M.S. (1958). Antioxidant determinations by the use of a stable free radical. Nature.

[18] Brand-Williams W., Cuvelier M.E., Berset C. (1995). Use of a free radical method to evaluate antioxidant activity. LWT.

[19] Re R., Pelegrini N., Proteggente A., Pannaca A., Yang M., Rice-Evans C. (1999). Antioxidant activity applying and improved ABTS radical cátiondescolorization assay. Free Radic. Biol. Med..

[20] Jardini F.A., Mancini-Filho J. (2007). Antioxidant activity evaluation of different polarities extract by pulps and seeds of pomegranate *Punica granatum*, L.. Rev. Bras. Cienc. Farm..

[21] Dobias P., Pavlíková P., Adam M., Eisner A., Benová B., Ventura K. (2010). Comparison of pressurised fluid and ultrasonic extraction methods for analisys of plant antioxidants and their antioxidant capacity. Cent. Eur. J. Chem..

[22] Mira N.V.M., Barros R.M.C., Schiocchet M.A., Noldin J.A., Lanfer-Marquez U.M. (2008). Extração, análise e distribuição dos ácidos fenólicos em genótipos pigmentados e não pigmentados de arroz (*Oryza sativa* L.). Ciênc. Tecnol. Aliment..

[23] NCCLS (2003). Performance Standards for Antimicrobial Disk Susceptibility Tests.

[24] Maia-Araújo Y.L.F., Mendonça L.S., Orellana S.C., Araújo E.D. (2011). Comparação entre duas técnicas utilizadas no teste de sensibilidade antibacteriana do extrato hidroalcoólico de própolis vermelha. Sci. Plena.

[25] NCCLS (2003). Methods for Dilution Antimicrobial Susceptibility Tests for Bacteria that Grow Aerobically.

[26] Ostrosky E.A., Mizumoto M.K., Lima M.E.L., Kaneko T.M., Nishikawa S.O., Freitas B.R. (2008). Métodos para avaliação da atividade antimicrobiana e determinação da concentração inibitória mínima (CIM) de plantas medicinais. Rev. Bras. Farmacogn..

[27] Ramalho V.C., Jorge N. (2006). Antioxidantes utilizados em óleos, gorduras e alimentos gordurosos. Quim. Nova.

[28] Hossain M.B., Barry-Ryan C., Martin-Diana A.B., Brunton N.P. (2010). Effect of drying method on the antioxidant capacity of six Lamiaceae herbs. Food Chem..

[29] Fawole O.A., Amoo S.O., Ndhlala A.R., Light M.E., Finnie J.F., Staden J.V. (2010). Anti-inflammatory, anticholinesterase, antioxidant and phytochemical properties of medicinal plants used for pain related ailments in South America. J. Ethnopharmacol..

[30] Akinmoladum A.C., Obuotor E.M., Farombi E.O. (2010). Evaluation of antioxidant and free radical scavenging capacities of some Nigerian Indigenous medicinal plants. J. Med. Food.

[31] Melo J.G., Araújo T.A.S., Castro V.T.N.A., Cabral D.L.V., Rodrigues M.D., Nascimento S.C., Amorim E.L.C., Albuquerque L.P. (2010). Antiproliferative activity, antioxidant capacity and tannin content in plants of semi-arid northeastern Brazil. Molecules.

[32] Gulati V., Harding I.H., Palombo E.A. (2012). Enzyme inhibitory and antioxidant activities of traditional medicinal plants: Potential application in the management of hyperglycemia. BMC Complement. Altern. Med..

[33] Oliveira A.M.C. (2011). Caracterização Química, Avaliação da Atividade Antioxidante *in vitro* e Atividade Antifúngica de Pimentas do Gênero *Capsicum* spp.. Master’s Thesis.

[34] Song F.L., Gan R.Y., Zhang Y., Xiao Q., Kuang L., Li H.B. (2010). Total phenolic contents and antioxidant capacities of selected Chinese medicinal plants. Int. J. Mol. Sci..

[35] Botterweck A.A.M., Verhagen H., Goldbohm R.A., Kleinjans J., Brandt P.A.V.D. (2000). Intake of butylated hydroxyanisole and butylated hydroxytoluene and stomach cancer risk: Results from analyses in the Netherlands cohort study. Food Chem. Toxicol..

[36] Yeh C.C., Chung J.G., Wu H.C., Li Y.C., Lee Y.M., Hung C.F. (2000). Effects of butylated hydroxyanisole and butylated hydroxytoluene on DNA adduct formation and arylamines *N*-acetyltransferase activity in PC-3 cells (human prostate tumor) *in vitro*. Food. Chem. Toxicol..

[37] Trindade R.A., Lima A., Andrade-Wartha E.R., Silva A.M.O., Mancini-FIlho J., Villavicencio A.L.C.H. (2009). Consumer’s evaluation of the effects of gamma irradiation and natural antioxidants on general acceptance of frozen beef burger. Radiat. Phys. Chem. Oxf. Engl. 1993.

[38] Beddows C.G., Jagait C., Kelly M.J. (2000). Preservation of α-tocopherol in sunflower oil by herbs and spices. Int. J. Food Sci. Nutr..

[39] Broinizi P.R.B., Andrade-Wartha E.R.S., Silva A.M.O., Novoa A.J.V., Torres R.P., Azeredo H.M.C., Alves R.E., Mancini-Filho J. (2007). Avaliação da atividade antioxidante dos compostos fenólicos naturalmente presentes em subprodutos do pseudofruto de caju (*Anacardium occidentale* L.). Cienc. Tecnol. Aliment..

[40] Ranilla L.G., Kwon Y.-I., Apostolidis E., Shetty K. (2010). Phenolic compounds, antioxidant activity and *in vitro* inhibitory potential against key enzymes relevant for hyperglycemia and hypertension of commonly used medicinal plants, herbs and spices in Latin America. Bioresour. Technol..

[41] Nazemiyeh H., Kazemi E.M., Zare K., Jodari M., Nahar L., Sarker S.D. (2010). Free radical scavengers from the aerial parts of *Euphorbia petiolata*. J. Nat. Med..

[42] Duarte M.C.T. (2006). Atividade antimicrobiana de plantas medicinais e aromáticas utilizadas no Brasil. Multiciência.

[43] Upadhyay B., Singh K.P., Kumar A. (2010). Etho-medicinal, phytochemical and antimicrobial studies of *Euphorbia tirucalli* L.. J. Phytol..

[44] Vale V.V., Orlanda J.F.F. (2011). Atividade antimicrobiana do extrato bruto etanólico das partes aéreas de *Euphorbia tirucalli* Linneau (Euphorbiaceae). Sci. Plena.

[45] Falleh H., Ksouri R., Chaieb K., Bouraoui N.K., Trabelsi N., Boulaaba M., Abdelly C. (2008). Phenolic composition of *Cynara cardunculus* L. organs, and their biological activities. BMC Pharmacol. Toxicol..

